# Flipped classroom combined with case-based learning is an effective teaching modality in nephrology clerkship

**DOI:** 10.1186/s12909-021-02723-7

**Published:** 2021-05-15

**Authors:** Fuye Yang, Wanbing Lin, Yan Wang

**Affiliations:** 1 Department of Nephrology, The Second Affiliated Hospital, Zhejiang University School of Medicine, 31009 Hangzhou, Zhejiang P.R. China; 2 College of Life Science, Xinyang Normal University, 464000 Xinyang, Henan P.R. China

**Keywords:** Flipped classroom, Traditional lecture-based teaching, Nephrology, Medical Education

## Abstract

**Background:**

The flipped classroom (FC) is recognized as an effective teaching approaches by emphasizing on the development of high-order abilities; however, the implementation of FC has not been well explored in nephrology education. The present study aims to investigate the efficacy of FC in teaching nephrology via comparing with the traditional lecture-based teaching (LBT).

**Methods:**

Sixty-two medical clerkship students at Zhejiang University School of Medicine were equally allocated into either LBT or FC group demographically matched. The glomerular diseases module was chosen for the teaching content. Students from the FC group were required to study the pre-class materials in annotated PPT format in advance. In the class, case-based learning (CBL) was employed, students encountered the related clinical cases and participated in the face-to -face discussion. Students from the LBT group attended a didactic lecture during the class. Quiz and questionnaires were performed to assess the efficacy of FC versus LBT.

**Results:**

Participants from the FC group performed better in the quiz than those from the LBT group with higher total scores (78.06 ± 2.515 vs. 65.16 ± 3.209, mean ± SEM), particularly the scores of the case analysis-related questions (35.81 ± 1.657 vs. 27.42 ± 1.910, mean ± SEM). In the survey, more students considered FC beneficial to comprehension, critical thinking, patient management and team work as compared with LBT. Meanwhile, more participants agreed increased in-class pressure in FC than in LBT.

**Conclusions:**

This study shows the positive impact of FC combined with CBL approach on nephrology education and provides an alternative pre-class and in-class format for the FC implementation.

**Supplementary Information:**

The online version contains supplementary material available at 10.1186/s12909-021-02723-7.

## Background

Nephrology is a complex subject and widely viewed as highly specialized field focusing on rare and severe diseases [1, 2]. Great advances have been achieved in nephrology in recent years, including the newly identified mechanism of the pathogenesis, a more profound understanding of the pathological changes, the updated diagnostic criteria, and the evolving novel therapeutic strategies of renal diseases. Teaching nephrology itself is a demanding, complex and often frustrating task. The recent advancements in nephrology but the relative lag of nephrology teaching mode further raised a challenge to nephrology educators. To fulfill the requirements to the current medical education system, nephrology education should focus on fostering students’ ability to solve complicated clinical problems [3–5].

The traditional instructional approach is the major educational mode in medicine education in China, which is based on the lecture-based teaching (LBT) with the teacher centered, emphasizing the delivery of knowledge and concepts [6]. The typical scenario of LBT is one that the teacher explains the theoretical knowledge and the students listen, take notes, and passively accept the knowledge. This approach benefits the memorization of facts within the limited period, while little attention is paid to problem-solving, critical thinking, teamwork and self-motivated learning [7]. Medicine including nephrology is a practical science, theoretical knowledge is only the basis basing on which to solve the complex clinical problems. Thus, the traditional teaching modality couldn’t fulfill the need of modern medicine and has been shown to be less effective than other teaching strategies in high-order learning abilities [8, 9].

The flipped classroom (FC) represents an ongoing paradigmatic shift in education from teacher-centered passive instructional strategies to student-centered active learning strategies [10]. Active learning has been defined as “any instructional method that engage students in the learning process” [11]. The FC reverses the traditional educational framework of LBT, where the students are given faculty-provided instructional content to review outside the classroom and take part in face-to-face interactive learning based on their preparatory work under the instructor’s guidance in the classroom with the goal of facilitating higher order learning of the materials. The FC is growing in popularity in education, especially the medical education [9, 12–18].

The FC method has shown greater academic achievement than traditional LBT, and this fact has been more evident in recent years [9]. Literature has reported the effectiveness of the FC in various health sciences education including pharmacology, radiology, emergency medicine, dermatology, physiology, and other subjects in the past decades [13–18]. The FC approach has also been extended to the medical clerkship and pre-vocational training with encouraging outcomes [19–22]. It has been also demonstrated that the use of FC improves board scores, increases resident satisfaction and enhances the self-perceived competence of the material [23, 24]. However, the implementation of FC in nephrology education has not been well explored. The aim of this study was to evaluate the students’ learning outcomes and enjoyment of the pedagogies between traditional LBT and FC in nephrology clerkship.

## Methods

### Participants

The present study was performed from July 2018 to December 2019. A total of 62 fourth-year medical students majoring in clinical medicine who have studied medical lessons for three years at Zhejiang University School of Medicine were voluntarily enrolled at the Second Affiliated Hospital of Zhejiang University School of Medicine. They had previously attended the nephology lectures by the same instructors before the interventions. The lectured content of glomerular diseases, including nephrotic syndrome and glomerulonephritis was delivered in 4 teaching hours. All the participants have also joined the same practical classes beforehand. Before the enrolment into the study, all students have taken the Internal Medicine Examination covering nephrology and other internal medicine subjects, which provided the assessment of the students’ previous performance. Based on these conditions, the participants were allocated into either FC (*n* = 31) group or LBT (*n* = 31) group with gender matched (Table 1). The research was approved by the Institutional Review Board and Ethics Committee of the Second Affiliated Hospital of Zhejiang University School of Medicine.

**Table 1:** Demographic information of participants in the study

Number of participants (percentage)			
		**FC** (*n* = 31)	**LBT** (*n* = 31)
**Gender**			
Male	19 (61.29 %)		21 (67.74 %)
Female	12 (38.71 %)		10 (32.26 %)

FC: flipped classroom group; LBT: lecture-based teaching group

### Study design

**Fig. 1:**
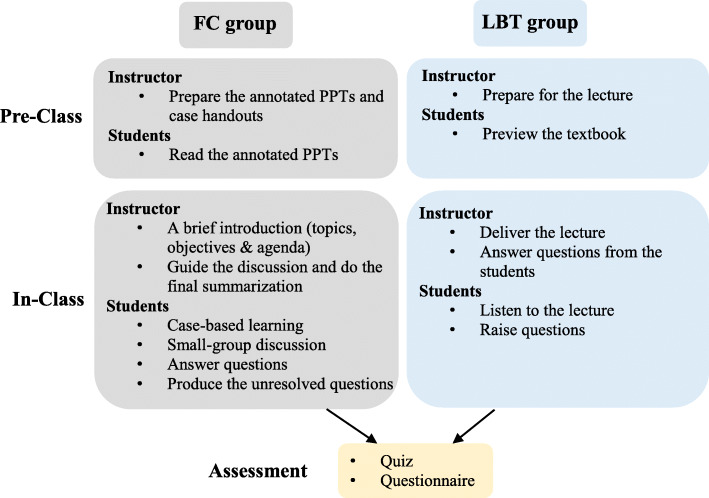
Schematic demonstration of the process of teaching activities. In LBT model, students are exposed to new materials in class through lecture delivered by the instructor. In contrast, students in FC model are first exposed to the material prior to class through faculty-generated resources (annotated PPT files) and involved in active case-based learning during the class. LBT, traditional lecture-based teaching; FC, flipped classroom

We chose the module of glomerular diseases to apply the teaching approaches in this study. The study was carried out following the flowchart as demonstrated in Fig. 1. Briefly, FC proceeded as follows: participants were provided with instructor-generated lecture notes in annotated PPT format and required to view the materials on his/her own time one week before the class. The class session started with a brief introduction of the topic, learning objectives and class agenda by the instructor. An active learning format is utilized where students encountered the real clinical cases and were challenged to take turns interpreting and discussing the cases which are representative of the glomerular diseases covered in the preassigned reading materials. The attending nephrologist provided guidance and feedback as the students interpreted the representative cases, pointing out characteristic and atypical findings for each case as well as providing clinical correlation. Finally, the instructor made the summary for the class and went over the tough questions raised by students during discussions. LBT run as follows: participants were encouraged to preview the related textbook or reference materials prior to the class and then attended a didactic lecture followed by a question-and-answer session in class where the same content as provided for the FC group was covered and aligned with the same learning objectives as those in FC group.

### Data evaluation and statistical analysis

To evaluate the learning outcomes, students were required to complete a post-class quiz related with the glomerular diseases when they had finished the study of this module (Additional file 1). All items in the quiz were A2-type questions aiming to evaluate both basic theoretical knowledge and clinical case analysis ability based on the Bloom’s Taxonomy of cognitive activities with “remember” and “understand” categories collapsed into “basic theoretical knowledge” and items in any of the other categories considered “clinical case analysis” [25, 26]. The scores were calculated and compared between the two groups by an independent samples *t*-test.

The participants from both groups were also required to complete a questionnaire with questions on their perception of and experience with the teaching models (items covering both positive and negative aspects) as well as their self-evaluations using a three-point Likert-type scale (− 1, disagree; 0, neutral; 1, agree) (Additional file 2). The questionnaire was modified from Paul Ramsden’s Course Experience Questionnaire and Biggs’ Study Process questionnaire with verified reliability and validity [27–29].

All methods were carried out in accordance with relevant guidelines and regulations.

## Results

### Students’ performance in the post-class quiz

**Fig. 2:**
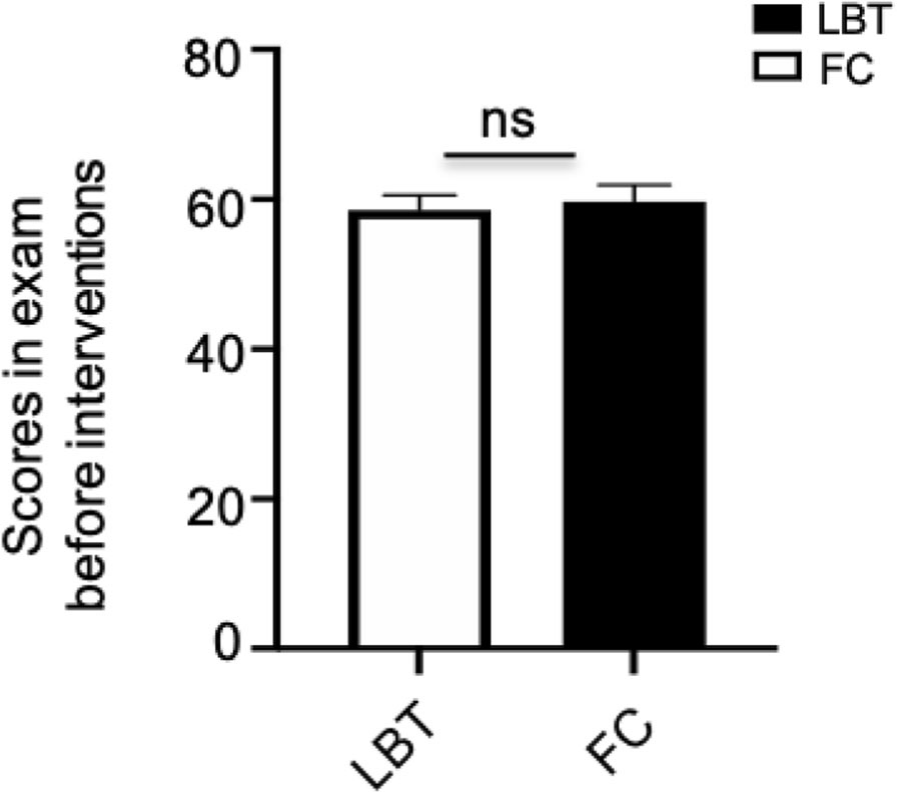
Comparison of students’ performance in the exam of Internal Medicine before the interventions. The students’ scores in the exam of Internal Medicine covering nephrology and other subjects such as cardiology, endocrinology, hematology, respiratory diseases and gastroenterology were assessed between the FC group and LBT group before the interventions. An independent samples *t*-test was used to compare the differences between these two groups. *t* = 0.3850 (*df* = 60), **p = 0.7016. Data were presented as mean ± SEM. NS: not significant. LBT, lecture-based teaching; FC, flipped classroom

**Fig. 3:**
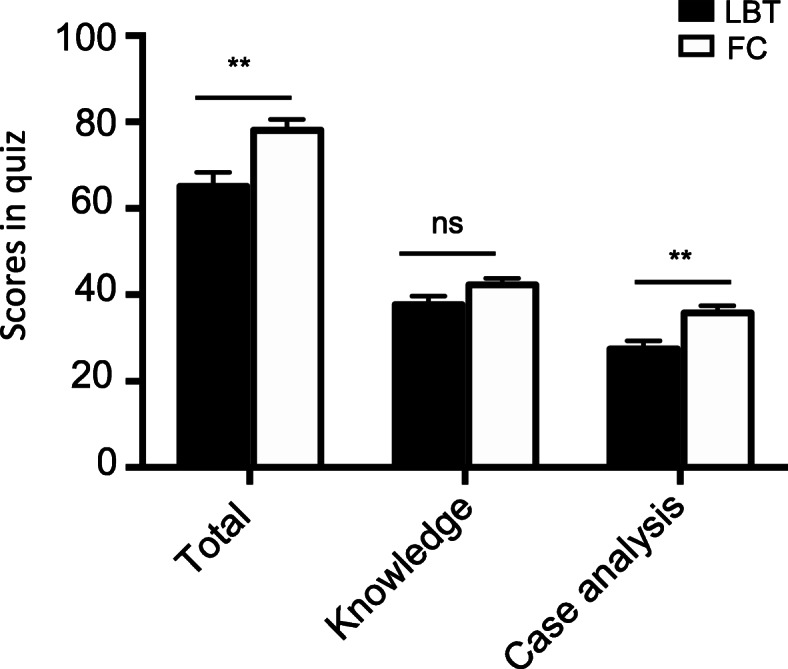
Comparison of students’ quiz scores between the FC group and LBT group after the classroom. Total, knowledge-related and case analysis-related questions were scored respectively. An independent samples *t*-test was used to compare the differences between the two groups. Data were presented as mean ± SEM. In total questions, *t* = 3.165 (*df* = 60), **p = 0.0024,; in knowledge-related questions, *t* = 1.795 (*df* = 60), *p* = 0.0776; in case analysis-related questions, *t* = 3.316 (*df* = 60), **p = 0.0016. NS: not significant. LBT, lecture-based teaching; FC, flipped classroom

A total of 62 students were enrolled in the study, including 31 students assigned to the FC group and 31 students assigned to the LBT group. The gender of the two groups is matched (Table 1.). To assess whether the previous performance of the students from each group was comparable, the results of Internal Medicine examination taken just before their entry into the clerkship were analysed as shown in Fig. 2, which demonstrated no significant difference between these two groups (*p* = 0.7016). The class attendance rates of both groups were 100 %. The efficacy of teaching modalities on the students’ performance were assessed by the post-class quiz which was conducted when the study of the glomerular disease module was finished. The data showed that students in FC group had higher average scores than those in LBT group (78.06 ± 2.515 vs. 65.16 ± 3.209, 95 % CI: 4.748 to 21.06, *p* = 0.0024). Further analysis revealed that there was no difference in scores related with basic theoretical knowledge between the two groups (42.26 ± 1.518 vs. 37.74 ± 2.006, 95 % CI: -0.5152 to 9.54, *p* = 0.0776), while higher score regarding with the clinical analysis ability was observed in the FC group than the traditional LBT group (35.81 ± 1.657 vs. 27.42 ± 1.910, 95 % CI: 3.328 to 13.45, *p* = 0.0016) (Fig. 3). These findings suggested that both LBT and FC were suitable for the delivery of basic theoretical knowledge, but in case of training for the higher level of cognitive abilities, FC presented greater advantages than LBT.

### Students’ self-perceived competence and satisfaction survey

**Fig. 4:**
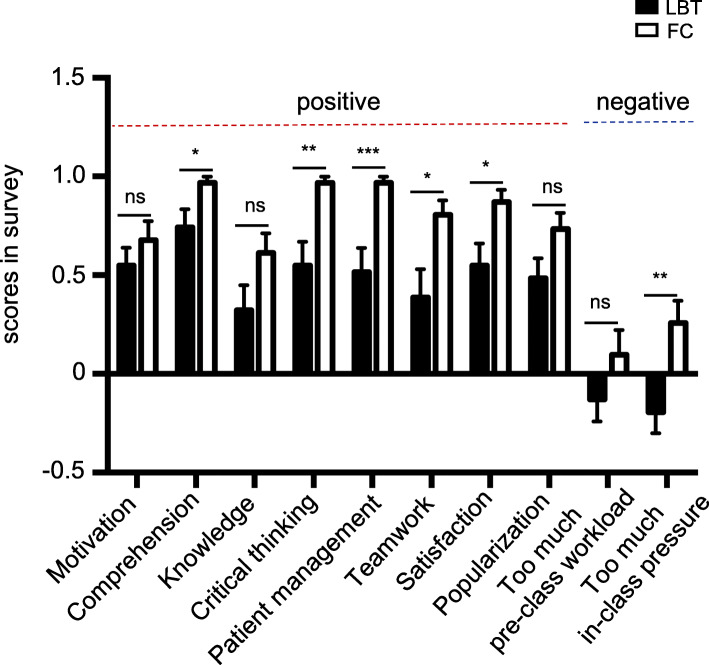
Comparison of students’ self-perceived competence and perspectives on the teaching modality experienced between the FC group and LBT group. Students’ answers to the survey questions were quantified using a three-point Likert scale (-1, disagree; 0, neutral; 1, agree). Data presented indicate the mean score ± SEM. *p < 0.05, **p < 0.01. ***p < 0.001. NS: not significant. LBT, lecture-based teaching; FC, flipped classroom

Next, students’ self-perceived competence was compared after taking the FC and LBT classes respectively. The response rates for the questionnaire of both groups were 100 %. As shown in Fig. 4, more students agreed that the FC could help improve their abilities of comprehension (*p* = 0.0245), critical thinking (*p* = 0.0014) and patient management (*p* = 0.0007) compared with the LBT, while there was no difference in learning fundamental knowledge (*p* = 0.0765) between two groups. This was consistent with the students’ performance in the quiz where students from the FC group performed better in high order cognitive abilities than those from the LBT group. Meanwhile, more students agreed that the FC could benefit their teamwork (*p* = 0.0117). These positive perspectives produced a higher rate of satisfaction with FC than LBT (*p* = 0.0142). In contrast, more in-class pressure was considered by students from the FC group than LBT group (*p* = 0.0054). Although pre-class study is the critical component for FC, there was no significant difference in the students’ opinion about the pre-class workload (*p* = 0.1835) (Fig. 4).

## Discussion

With the increase in the need for nephrology care and deeper insights into the functioning of the kidney, traditional lectures do not align well with the current requirement for nephrology education to prepare our medical students to practice quality clinical care and undertake research to understand renal physiology and pathophysiology in future [1, 3–5]. Recently, there are emerging alternative approaches to the traditional didactic lecture, including the FC and CBL, which focus on improving the medical students’ critical thinking and solving clinical practical problems [7, 8, 10, 11]. These active-learning approaches are gaining more and more popularity in medical education. While the efficacy of FC over LBT seems to rest on subjects and specific settings [30]. In the present study, we implemented FC with CBL combined in the class activities in nephrology clerkship students and compared the students’ perspectives and performance with those experienced LBT teaching modality, which was a novel attempt and provided insights for teaching of other modules in nephrology.

We chose glomerular diseases module as the topic in our study for two reasons. First, the glomerular diseases are among the leading causes of end stage renal diseases, thus this module is one of the most import chapters and always comes first in nephrology teaching for different levels of learners. Second, they share similar symptoms and signs but possess differential pathogenesis and pathological patterns, which is complicated and energy-spending for students to understand and master. Therefore, it is necessary to explore an effective instructional approach.

The present study investigated into the efficacy of the FC combined with CBL approach in a clinical education setting of glomerular diseases. To our knowledge, this modality has not been well studied in nephrology education. Here we demonstrated that the FC group outscored LBT group in the post-class quiz (Fig. 3). Further analysis revealed that the improvement in total scores in FC group was largely related with the case-analysis type of questions, while no significant difference was observed in scores of theoretical knowledge-related questions between the two groups. These results were consistent with the findings from the previous FC studies on subjects other than nephrology that the FC approach improves students’ skills and competence and fosters the high-order cognitive abilities [14, 22]. This advantage of FC may be attributed to several aspects during the preparation and implementation of the course [10, 12]. In the pre-class session, FC provides self-paced pre-class learning and students can use their time more efficiently. During the class, FC emphasizes on the high levels of cognitive abilities by encouraging students to utilize what they have learned to solve the more complex problems. This output process is involved in not only the knowledge and comprehension but also the ability of application, analysis and synthesis. Thus, FC offers a sequential and gradual learning process which bridges the gap between pre-class learning of foundational knowledge and in-class training of application and problem-solving abilities [10, 12].

Meanwhile, we also compared students’ self-perceived competency and perceptions of the teaching modalities between the two groups. More students from the FC group considered the course to be helpful to improve their overall comprehension of the content, critical thinking and patient management abilities than those from the LBT group (Fig. 4). The student’ responses were consistent with their performance in the quiz that the FC group generally outperformed the LBT group. Similar to our finding, previous studies have shown that students showed more positive opinion on the FC than the traditional LBT approach in various medical subjects, including pharmacology, ophthalmology, emergent medicine and radiology [14, 22]. The session of face-to-face interactions in the FC group was also considered helpful to build up the confidence in their teamwork in contrast to the LBT group. Thus, more students from the FC group showed satisfaction with the course than those from the LBT group. These positive feedbacks from the students should be particularly encouraging to educators to consider applying the FC model to other nephrology modules since it is evidently effective in improving the wide-spectrum cognitive abilities of students.

Interestingly, although participants in FC group performed better as a whole, some of them gave negative feedback about the in-class pressure. This negative feedback might be partly attributed to the learner’s reluctance to take a more active role in the new teaching modality. The FC model is characterized by the student-centered, active learning, which can be challenging to students who are accustomed to the passive learning in medical school. The requirement of active involvement in the in-class presentation and discussion was considered an extra burden by these students. The burden and pressure may thus compromise the satisfaction with the course.

There are two limitations in this study. One is that we did not extend the study to the after-class work but just focused on pre-class and in-class activities, while after-class activities help in reinforcing and optimizing the prior learning with ongoing practice. Complexity, spacing, and time constraints are critical factors influencing the implementation of after-class work, which can be achieved in a structured way through additional programs. Another limitation is that we assessed only the short-term results of this teaching approach. Further studies are guaranteed to investigate the long-term benefits of this teaching approach to residency training and even career development.

## Conclusions

In summary, FC combined with CBL represents an effective and flexible approach in medicine education and can be tailored to meet the various education situations. Further studies with elaborated design to optimize FC in terms of specific subjects, student’s workload, optimal strategies and the evaluation system could help further advance the impact and effectiveness of FC.

## Supplementary information

Additional file 1 Supplementary Quiz

Additional file 2 Supplementary Survey

## Data Availability

The related materials including the teaching materials, forms, quiz and questionnaire are kept in hard and/or soft copies in the Department of Nephrology Zhejiang University School of Medicine Second Affiliated Hospital. The datasets generated during and analysed during the current study are available from the corresponding author on reasonable request.

## Abbreviations

FC: Flipped classroom
LBT: Lecture-based teaching
