# Supplementary material for: Flipped classroom combined with case-based learning is an effective teaching modality in nephrology clerkship
Source: BMC Med Educ. 2021 May 15;21:276. doi: 10.1186/s12909-021-02723-7 (PMC8122572; doi:10.1186/s12909-021-02723-7)
Supplement: Supplementary file 1 — Supplementary Quiz [file 12909_2021_2723_MOESM1_ESM.pdf]

Quiz

Student ID: _______________ Group: _______________ Date: ________________

1. A 60-year-old male was admitted for "5 days of edema". Physical examination is unremarkable except edema in eyelids and bilateral lower extremities. Laboratory test: urinalysis: protein (+++), blood biochemistry: albumin 26.7 g/L， creatinine 99 µmol/L. Anti-phospholipase A2 receptor antibody is positive. Anti-nucleus antibodies， serum light chain, and cancer-related biomarkers are unremarkable. Renal biopsy reveals mild mesangial cell and mesangial matrix proliferation, and rigid appearance of capillary loop with opened lumen under light microscope; thickened glomerular basement membrane with structure of "spikes and holes" under electron microscope and granular deposits of IgG (+++) and IgM (+). What is the most likely diagnosis for this patient?
   A. Minimal change disease
   B. Mesangial proliferative glomerulonephritis
   C. Membranous nephropathy
   D. Membranoproliferative glomerulonephritis
   E. Focal segmental glomerulosclerosis
2. A 36-year-old male presented with 6 days of gross hematuria, reduced urine output, nausea, and vomiting. Physical examination: BP168/92mmHg, bilateral pitting edema in lower extremities. Urinalysis: protein (++)， RBC 240/HPF; Hb 89g/L; Serum creatinine 490 µmol/L. Levels of C3 and C4 are within normal range. Ultrasound reveals enlarged kidney size. The patient denied any disease histories including hypertension or diabetes. What is the most likely diagnosis for this patient?
   A. Acute pyelonephritis
   B. Postinfectious glomerulonephritis
   C. Rapidly progressive glomerulonephritis
   D. Acute interstitial nephritis
   E. Chronic glomerulonephritis with acute aggravation

3. The typical electron microscopic findings on renal biopsy of a 15-year-old boy with nephrotic syndrome who responds well to corticosteroid therapy is.
   A. Fusion of foot processes
   B. Subepithelial electron dense deposit
   C. Duplication of glomerular capillary basement membranes
   D. Irregular thickening of the glomerular basement membranes
   E. Mesangial cell proliferation
4. A 44-year-old female was admitted for two weeks of edema. BP: 135/80mmHg， generalized pitting edema. Urinalysis: protein (++++), RBC (++), serum creatinine 122 μmol/L. Serum albumin 26 g/L. Renal biopsy reveals hypercellularity of mesangial and inflammatory cells, evident mesangial matrix proliferation and the interposition of mesangial cells between glomerular epithelial cells and basement membrane. Which is the most likely diagnosis?
   A. Membranous nephropathy
   B. Mesangial proliferative glomerulonephritis
   C. Mesangiocapillary glomerulonephritis
   D. Endocapillary proliferative glomerulonephritis
   E. IgA nephropathy
5. A 14-year-old girl was hospitalized for edema. She noticed periorbital edema two weeks ago, which became generalized 3 days ago. Laboratory tests show urine protein (++++)， urine albumin/creatine: 8739 mg/g, serum albumin 13.1g/L, total cholesterol 12.45 mmol/L, serum creatinine 63 μmol/L. It is unremarkable on light microscopy and immunofluorescence but diffuse podocyte foot process effacement on electron microscopy. Which is the preferred regimen for the patient?
   A. Cyclosporine A
   B. Corticosteroid combined with cyclosporine A
   C. Cyclophosphamide
   D. Corticosteroid combined with cyclophosphamide
   E. Corticosteroid
6. A 30-year-old male was hospitalized for heavy proteinuria and pitting edema and diagnosed with nephrotic syndrome. Renal biopsy reveals minimal change

disease. Thus, the patient received oral prednisone 60mg/d. On week 3 of therapy, the patient had elevated serum creatinine level with aggravated heavy proteinuria and edema. Which is the most likely situation that you may consider?

A. Infection
B. Renal vein thrombosis
C. Corticosteroid pulse therapy
D. Cytotoxic medication
E. Disease relapse

7. A 29-year-old male with 10 years of type 1 diabetes complains of recurrent edema for 1 year. BP: 158/92mmHg, urinalysis: protein (+++), RBC 2/HPF, glucose (+). Urine albumin/creatinine 6949 mg/g, serum creatinine 84 μmol/L. What is the typical pathological change for the patient?
    A. Diffuse glomerulosclerosis
    B. Mesangial and endothelial proliferation
    C. Thickened glomerular basement membrane with spike-like appearance
    D. Nodular glomerulosclerosis
    E. Endocapillary proliferation
8. A 19-year-old female was admitted for fatigue and edema. She complains of recurrent oral ulcers and alopecia. Physical examination reveals skin rash, pale face appearance and bilateral edema in lower extremities. Urinalysis: protein (++), RBC (++), hemoglobulin 89 g/L, serum creatinine 152 μmol/L, serum C3 0.45 g/L, anti-dsDNA 146.5 IU/ml. What is the most likely diagnosis for the patient?
    A. Acute glomerulonephritis
    B. Henoch-Schonlein purpura nephritis
    C. Rapidly progressive glomerulonephritis
    D. Nephrotic syndrome
    E. Lupus nephritis
9. A 25-year-old female complains of abrupt edema for 5 days. Laboratory tests show urine protein (++++), urine RBC 2/HPF, serum albumin 15.1 g/L, total cholesterol 12.45 mmol/L, serum 63umol/L. Renal biopsy reveals mild

mesangial proliferation with expanded inter-tubular space. The adhesion of capillary loop to Bowman's capsule is observed in 1 of 18 glomeruli. It is unremarkable for immunofluorescence. Diffuse effacement of podocyte foot process is evident under electron microscope. Which is the most likely diagnosis for the patient?

A. Membranous nephropathy
B. Mesangial proliferative glomerulonephritis
C. Membranoproliferative glomerulonephritis
D. Focal segmental glomerulosclerosis
E. Minimal change disease

10. A 31-year-old female presents with one day of gross hematuria. She had a fever and cough 3 days ago. Physical examination is unremarkable. Urine RBC 331/μL which are predominantly of smaller and dysmorphic erythrocytes， urine protein (+), urine WBC 2/μL. Serum creatinine 72 μmol/L. Which is the most likely diagnosis of the patient?

A. Acute glomerulonephritis
B. Rapidly progressive glomerulonephritis
C. IgA nephropathy
D. Urinary tract infection
E. Nephrotic syndrome
