# Supplementary material for: Flipped classroom combined with case-based learning is an effective teaching modality in nephrology clerkship
Source: BMC Med Educ. 2021 May 15;21:276. doi: 10.1186/s12909-021-02723-7 (PMC8122572; doi:10.1186/s12909-021-02723-7)
Supplement: Supplementary file 2 — Supplementary Survey [file 12909_2021_2723_MOESM2_ESM.pdf]

Survey

Student ID: ________________________ **Group:** ______________________ **Date:** ________________________

**Items**	**Opinion**		
	**disagree**	**neutral**	**agree**
The course helps to enhance my motivation of learning			
The course benefits the comprehension of the content			
The course promotes the memorization of the knowledge			
The course helps to develop the ability of critical thinking			
The course helps to develop the ability of patient management			
The course promotes the teamwork			
I feel satisfied with the course as a whole			
The course should be popularized in other subjects			
The course gives me too much pre-class workload			
The course gives me too much in-class pressure			

Note: This survey adopted the three-point Likert-type scale (−1, disagree; 0, neutral; 1, agree).
